# The Incidence and Risk Factors of Acute Kidney Disease after Total Knee Arthroplasty with Early Postoperative Volume Supplement

**DOI:** 10.1155/2018/8718545

**Published:** 2018-07-17

**Authors:** Kuan-Ting Wu, Chung-Yang Chen, Bradley Chen, Jun-Wen Wang, Po-Chun Lin, Shih-Hsiang Yen

**Affiliations:** ^1^Department of Orthopaedic Surgery, Kaohsiung Chang Gung Memorial Hospital, Taiwan; ^2^Institute of Public Health, National Yang Ming University, Taipei, Taiwan; ^3^Chang Gung University, College of Medicine, Taoyuan, Taiwan

## Abstract

**Background:**

Etiology of acute kidney disease (AKD) after total knee arthroplasty (TKA) was considered as multifactorial. However, the role of early postoperative volume supplement in AKD rate has not been investigated. The purpose of this study was to evaluate the incidence and risk factors of AKD in patients with early volume supplement following TKA.

**Methods:**

This was a retrospective study with 458 patients who underwent unilateral TKA. All the patients received 6% tetrastarch, 7.5ml/kg, early in the postoperative period. Postoperative AKD was defined as the postoperative creatinine level ≥ 1.5 times compared with preoperative data. Potential variables associated with AKD were analyzed by multivariate logistic regression model to identify the AKD risk factors in TKA patients after early postoperative volume supplement.

**Results:**

The AKD rate was 3.3% (15 patients) in all patients. Age (OR = 1.09; P = .031) and* coronary artery disease (CAD)* (OR = 3.63; P = .034) were associated with increased risk of development of postoperative AKD. Other comorbidities as hypertension, diabetes, and CKD were not statistically significant risk factors.

**Conclusion:**

Our study demonstrated that age and* CAD* were independent risk factors of AKD in TKA patients. However, the common risk factors as hypertension, diabetes, and CKD were not significantly associated with AKD after TKA* if early postoperative supplement of tetrastarch is administered*.

## 1. Introduction

Total knee arthroplasty (TKA) is a well-established procedure with a promising outcome for aging patients who suffer from end-stage arthritis of the knee. Recently, a fast-track program has been popularized in hip and knee replacement surgery, the aim of which is to reduce hospital stay, enable quicker rehabilitation, and facilitate early recovery. The length of hospital stay has been reduced from 4–12 days to 1–3 days via this program [1, 2]. Orthopedic surgeons who perform lower-limb arthroplasty are dedicated to improving their surgical technique in addition to refining postoperative care in order to reduce postoperative complications and the length of hospital stay. However, patients with chronic kidney disease (CKD) have been reported to carry higher risks of cardiovascular disease, increased blood transfusion and postoperative infection, and acute kidney disease (AKD) after TKA [3]. The estimated blood loss after TKA ranges from 1000 to 1400 ml and results in a blood transfusion rate of 0–17.5% [4–7]. Anemia-related AKD via a hypotension episode or a decrease in oxygen-carrying capacity has been reported [8]. By the use of a pneumatic tourniquet to create a bloodless surgical field, the fibrinolytic system is activated, which increases blood loss after knee arthroplasty during the first few postoperative hours. An approximate 60–80% of total blood loss has been measured within the initial 8 hours after TKA [9]. Owing to increased bleeding during the postoperative period, the incidence of AKD after total joint arthroplasty has been reported to range from 2 to 15% [10–13], and the incidence of AKD in CKD patients is approximately 7–20% [11, 13, 14]. In addition, inadequate fluid resuscitation within 6 hours after deflation of the tourniquet may be considered as a risk factor for AKD after TKA, especially in patients with CKD.

Volume depletion results in hemodynamic change during the early recovery period and is considered a risk factor for AKD after total joint arthroplasty. Tetrastarch has been used as a volume expander, which increases the mean arterial blood pressure (MAP) and diastolic blood pressure (DBP) as compared with crystalloid solution in total hip arthroplasty; it also reduces molar substitution and polydispersity and has been demonstrated to result in fewer side effects on renal function in recent studies [16, 17].

Therefore, we hypothesized that prevention of volume depletion in the early postoperative period will decrease the incidence of AKD. The purpose of this study was to evaluate the effect of early volume supplement using tetrastarch colloid postoperatively on AKD in TKA patients.

## 2. Materials and Methods

This is a retrospective analysis of 481 primary TKA patients between June 2015 and July 2017 with prospectively collected data. The patients with end-stage renal disease (5 patients), rheumatoid arthritis with long-term steroid consumption (11 patients), or missing data (7 patients) were excluded. Finally, 458 patients were enrolled to our study. All the patients underwent primary unilateral TKA with cement fixation (Zimmer, NexGen LPS-Flex, Warsaw, USA) by a single surgeon. The study was approved by our Institutional Review Board. Indication for arthroplasty was end-stage arthritis of the knee. The postoperative protocol includes consecutive data of hemoglobin, serum creatinine, and estimated glomerular filtration rate (eGFR) for three days and the highest creatinine level was selected for the definition of renal injury. The blood transfusion criteria were set at Hb <7 g/dL or 8-9 g/dL if it is symptomatic in healthy patients or Hb <9 g/dL in patients with history of* coronary artery disease (CAD)*, stroke, or chronic kidney disease.* The definition of CAD is patients with history of stenosis of coronary arteries presenting as angina or old myocardial infarction with or without antiplatelet or anticoagulants prescription.* Prophylactic antibiotic therapy was intravenous injection of 1 g cefazolin preoperatively and every 8 hours postoperatively for 3 doses.

*Because the pneumatic tourniquet was used during the operation, the TKA procedure does not incur significant blood loss intraoperatively. The balanced electrolyte solution with Ringer's lactate was administrated depending on patients' blood pressure and body weight in the rate of 150-200 ml/hr if patients' body weight was lower than 80 kg and 200-250 ml/hr in patients who weigh more than 80 kg. Patients who had ESRD under hemodialysis, congestive heart failure (CHF) NYHA class III and IV, or allergy to hydroxyethyl starch would be excluded for the volume supplement. Since the patients with ESRD were excluded initially and the remaining 458 patients did not have severe CHF or allergic history*, all the 458 patients received 6% tetrastarch, HES 130/0.4 (Voluven®, Fresenius Kabi, Bad Homburg, Germany), 7.5ml/kg, as volume expander in early postanesthesia recovery room and additional 7.5 ml/kg was given on postoperative day 1. Crystalloid infusion with D5 0.225% saline or normal saline was given postoperatively with infusion rate of 60-80 ml/hr for 24 hours. 3000 mg of tranexamic acid was injected intra-articularly in* CAD* or cerebrovascular accident (CVA) patients or 1000 mg was injected intravenously in other patients during operation after cementing the knee prosthesis. Intravenous injection of 40 mg Dynastat® (Parecoxib; Pfizer, Pharmacia and Upjohn Company, USA) every 12 hours was prescribed for 2 days postoperatively. Indomethacin, Celecoxib, or Ultracet (Tramadol 37.5 mg and acetaminophen 325 mg, Janssen Korea Ltd.) was prescribed for postoperative pain control.

### 2.1. Outcome Measures

The maximum relative creatinine was identified and classified using the RIFLE criteria proposed by Acute Dialysis Quality Initiative Group (Table 1) [18] in which (1) RIFLE-R: eGFR decrease > 25%, serum creatinine >1.5 times; (2) RIFLE-I: eGFR decrease > 50%, doubling of serum creatinine; (3) RIFLE-F: eGFR decrease > 75%, tripling of serum creatinine; (4) RIFLE-L: complete loss of renal function for 4 weeks; and (5) RIFLE-E: end-stage renal disease. Increased postoperative serum creatinine value ≧ 1.5 times than preoperative value indicates risk of AKD.

Demographic data (age and gender), body mass index (BMI), comorbidities as CKD, diabetes mellitus (DM), hypertension (HTN),* CAD*, anesthesia type, ASA score, length of hospital stay, and operative time were collected. The relative serum creatinine was calculated by postoperative serum creatinine divided by preoperative serum creatinine and the estimated blood loss was calculated by formula proposed by* Good et al. [19]* and Nadler et al. [20]. Finally, we calculate the incidence of AKD, postoperative creatinine, relative creatinine, blood loss, and hospital stay and analyzed risk factors of AKD after early fluid supplements.

### 2.2. Statistical Analysis

The baseline characteristics are presented as mean and standard deviation. The Kolmogorov-Smirnov test was used to test the normality of the data. The possible risk factors for AKD, such as age, BMI, gender, preoperative eGFR, preoperative creatinine, preoperative anemia, blood transfusion, operative duration, total blood loss, CKD, coronary artery disease, hypertension, and diabetes mellitus, were analyzed by univariate binary logistic regression. Then, the predictors with a p value <.30 were analyzed with multivariate logistic regression using backward selection. A p value of less than 0.05 was considered significant. All statistical analyses were performed using SPSS software V.21 (SPSS Inc., Chicago, Illinois).

## 3. Results

Totally 458 cases were enrolled in the study, including 377 females (82.3%) and 81 males (17.7%) with a mean age of 70.52 years (range, 46-90 years). The average preoperative eGFR was 77.80±24.66 (mL/min/1.73m2). CKD accounts for 22.5% in this cohort (103/458). The average total estimated blood loss was 883.94±327.46 (ml) with 8.5% transfusion rate. The overall incidence of postoperative AKD after early volume replacement was 3.3% (*n* = 15). Of the 15 patients with AKD, 13 patients were AKR (RIFLE-R) and 2 patients were AKI ( RIFLE-I) (Table 2).

In the univariate logistic regression model, predictors as age (OR, 1.12; P = .006), length of stay (OR, 1.79; P = .036), and history of coronary artery disease (OR, 5.33; P = .004) were significantly associated with postoperative AKD. The preoperative eGFR, DM, HTN, and CKD were not associated with postoperative AKD (Table 3).

Multivariate logistic regression model disclosed that age (OR, 1.09; P = .031) and coronary artery disease (OR, 3.63; P = .034) were independently associated with increasing risk of postoperative AKD.

## 4. Discussion

In this study, we demonstrated a total incidence of 2.9% (13 patients) for AKR (RIFLE-R) and 0.4% (2 patient) for AKI (RIFLE-I). A retrospective study by Nowicka and Selvaraj [11] reported an incidence of AKD of 6.2% in 337 patients, including 48 who had CKD stage 3, after elective total hip and knee arthroplasty. In that study, the AKD incidence was 4.5% in the non-CKD patients and 16.3% in the CKD patients, and the researchers concluded that the risk of developing AKD was 4-fold after lower-limb arthroplasty if patients had a preoperative eGFR <60. Recently, Kimmel et al. reported an incidence of AKD in 425 patients following total joint arthroplasty (252 TKA and 173 THA) of 14.8% (63/425).[10] The AKD incidence in the TKA patients was 19.4% (49/252); the TKA patients were generally older in age and had more comorbidities such as hypertension, diabetes, cardiac disease, chronic obstructive pulmonary disease, and CKD as compared with the THA patients, which may have led to a higher AKD incidence after surgery. With regard to exposure to Celecoxib and ketorolac after joint arthroplasty, Warth et al. [13] disclosed an incidence of AKD of 4.8% in patients without significant preoperative renal impairment and 11.9% when patients were superimposed on CKD. To date, CKD has already been identified as a major risk factor for postoperative AKD.* In addition to CKD, other preoperative characteristics such as a high BMI, hypertension, diabetes mellitus, cardiac comorbidities, chronic lung disease, and peripheral vascular disease have been identified as risk factors for postoperative AKD in arthroplasty patients [10, 12, 21, 22].* Also, blood loss-related anemia and low blood pressure are significantly correlated with AKD, owing to the decreased blood flow to the kidneys [10, 12]. The incidence of AKD (3.9%) in our CKD patients was much lower than the 11.9% reported by Warth et al. [13] and 16.3% by Nowicka et al. [11] in CKD patients. We also found that eGFR and CKD were not associated with postoperative AKD after postoperative fluid supplement using tetrastarch.

Postoperative AKD is associated with multiple complications after lower-limb arthroplasty, which include pulmonary embolism, early infection, revision surgery, increased length of hospital stay, greater cost, and an increased rate of readmission [15, 23]. Owing to the high complication rate following AKD, the United Kingdom National Confidential Enquiry into Patient Outcome and Death (NCEPOD) has addressed the field of renal injury since 2009 and found that 20% of the mortality in AKD patients was predictable and avoidable. The implementation of “ABCDE,” which includes Address medications (NSAIDS, antihypertensive agents), Boost blood pressure, Calculate fluid balance, Dipstick urine, and Exclude obstruction, was proposed by Alister et al. to prevent renal injury following lower-limb arthroplasty [24]. Therefore, we focused on eliminating the impact of postoperative hypovolemia on AKD by using a fluid supplement in the early postoperative period. According to the theory of Benoni, 60–80% of the total blood loss occurs in the postoperative few hours after TKA [9]. In that situation, an approximate 600–1000 ml blood loss will occur during the postoperative period. Therefore, administration of a crystalloid supplement only will not allow build-up of a sufficient circulating volume for renal perfusion in the early postoperative period. Hartog et al. [25] demonstrated that colloids were more effective and safe for the resuscitation of a critically ill patient than crystalloids only.

Hydroxyethyl starch (HES) is a synthetic colloid composed of a nonionic starch derivative. The first generation of HES, hetastarch, had a high proportion of hydroxyethyl substitution with a 0.7 molar substitution. Owing to the high polydispersity, slow degradation of the molecule with >50% of the maximum value in the plasma after 24 hours [26] could lead to renal failure, and molecules were mostly accumulated in the proximal tubules of the kidney, resulting in osmotic nephrosis. The newer generation of HES, including pentastarch (0.5 molar substitution) and tetrastarch (0.4 molar substitution), reduces molar substitution and polydispersity and results in fewer side effects on renal function. The estimated plasma clearance of tetrastarch is at least 20-fold higher than that of hetastarch, and it does not accumulate in plasma in one dose or with multiple dose administration [27, 28]. Kancir et al. [16] conducted a randomized controlled trial and demonstrated that using tetrastarch 130/0.4 after THA did not result in any adverse effect on renal function. In a recent study, 118 patients who underwent hip arthroplasty under spinal anesthesia were randomized into 6% HES 130/0.4 or sodium lactate Ringer's solution treatment groups, and Zhang et al. reported that administration of HES was not associated with AKD [17].

This study disclosed two predictors independently associated with postoperative AKD: older age and* CAD*. In consistence with other studies regarding age in AKD, Zimmer et al. found that the risk of AKD increased by 7% with each year increase of age; Gharaibeh et al. [29] and Hassan et al. [12] also reported that older age was independently associated with postoperative AKD after total joint arthroplasty. In addition to the age, we revealed that* CAD* (OR, 3.63; P = .034) was significantly associated with postoperative AKD. As previous literature, Jafari et al. [21] and Gharaibeh et al. [29] found heart disease to be one of the major risk factors for postoperative AKD after joint arthroplasty.

In clinical situation, increased prostaglandin synthesis will occur in response to prolonged vascular constriction in kidney as a compensatory mechanism [30]. However, the compensatory mechanism might mitigate in patients with CKD or hypovolemia condition. For this reason, most studies [10, 12, 21, 22] demonstrated CKD as a major risk factor associated with postoperative AKD after total joint arthroplasty. Recently, Jämsä et al. [31] reported that the preoperative eGFR (OR, 0.98; P = .03) was independently associated with postoperative AKD in a large cohort study with 20575 patients undergoing arthroplasty. Furthermore, Gharaibeh et al. [29] identified CKD (OR, 4.6; P < .001) as a significant risk factor for postoperative AKD in 8949 patients in one institution following total hip arthroplasty. In this study, we did not find significant association of postoperative AKD with preoperative eGFR (OR, 1.01; P = .369) and CKD (OR, 1.26; P = .694) after early volume replacement with tetrastarch. In addition, the overall AKD rate (3.3%) was lower than what has been previously reported in studies in which our strategy was not employed [11, 13, 14].

We acknowledge some limitations of this study. First, this was a retrospective study, and data of urine output, daily input/output, and intraoperative blood pressure were not recorded in detail. Some studies have also demonstrated a delayed drop in renal function until one to two weeks postoperatively, but we only recorded the consecutive creatinine value on three days postoperatively owing to the short length of hospital stay. Second, even though we did not find a significant association of AKD with preoperative eGFR and CKD, the* post hoc* analysis of logistic regression model disclosed a power of 0.15 in eGFR and 0.08 in CKD. Thus, making a conclusion that preoperative eGFR and CKD were irrelevant in development of AKD postoperatively is premature. For more accurate data, further investigation using large cohort of patients and a longer follow-up for serum creatinine measurement is necessary.

## 5. Conclusion

Our study demonstrated a low incidence (3.3%) of postoperative AKD in TKR patients with early volume supplement. Older age and* CAD* were found to be independently associated with postoperative AKD. However, comorbidities such as hypertension, diabetes, and CKD are not significantly associated with AKD in our series after early tetrastarch therapy.

## Figures and Tables

**Table 1:** RIFLE criteria [15].

**AKI**	**Creatinine increase**	**GFR decreased **%	**Urine output**
Risk	1.5 times	>25	<0.5ml/kg/hr x 6hr
Injury	2 times	>50	<0.5ml/kg/hr x 12hr
Failure	3 times	>75	<0.5ml/kg/hr x 24hr or anuria x 12hr
Loss	Persistent ARF > 4 weeks		
ESRD	End stage		

**Table 2:** Demographic data.

Continuous variable	Mean ± SD	
Age (years)	70.52±6.89	
BMI (kg/m^2^)	28.29±4.64	
Preop. Cr (mg/dL)	0.85±0.31	
Preop. eGFR (mL/min/1.73m^2^)	77.80±24.66	
Preop. Hb (g/dL)	12.86±1.33	
OP time (min)	87.57±15.63	
Length of stay (days)	3.41±0.71	
Estimated blood loss (mL)	883.94±327.46	

Nominal variable	Number of patients (*N* = 458)	Percentage (%)

Gender (M/F)		
Male	81	82.3
Female	377	17.7
ASA		
1	7	1.5
2	279	60.9
3	171	37.3
4	1	0.2
Anesthesia		
Spinal	106	23.1
General	352	76.9
CKD	103	22.5
Diabetes	135	29.5
Hypertension	328	71.6
CAD	43	9.4
Transfusion rate	39	8.5
AKD	15	3.3

*∗*Preop.: preoperative.

*∗*Cr: creatinine.

*∗*eGFR: estimated glomerular filtration rate.

*∗*Hb: hemoglobin.

*∗*OP time: operative time.

*∗*CKD: chronic kidney disease; AKD: acute kidney disease.

*∗*CAD: coronary artery disease.

**Table 3:** Risk factors associated with postoperative AKD after early volume replacement.

Continuous variable	AKD	Non-AKD (*N* = 443)	Logistic regression				
	(*N* = 15)		Univariate		Multivariate		
			Odds ratio	P value	Odds ratio	95% CI	P value
Age (years)	75.33±7.02	70.35±6.84	1.12	.006	1.09	1.008-1.187	.031
BMI (kg/m^2^)	28.48±5.16	28.29±4.63	1.01	.875			
Preop. Cr (mg/dL)	0.80±0.28	0.86±0.31	0.53	.521			
Preop. eGFR (mL/min/1.73m^2^)	83.40±36.41	77.60±24.21	1.01	.369			
Preop. Hb (g/dL)	12.38±1.50	12.87±1.32	0.76	.157			
OP time (min)	87.60±15.82	87.56±15.64	1.00	.993			
Length of stay (days)	3.8±0.94	3.40±0.69	1.79	.036			
Estimated blood loss (mL)	851.50±381.35	885.04±325.93	1.00	.696			

Nominal variable							

Gender (M/F)							
Female	13	364	0.72	.667			
Male	2	79					
ASA			0.52	.197			
1		7					
2	7	272					
3	8	163					
4		1					
Anesthesia							
Spinal	2	104	1.99	.369			
General	13	339					
CKD							
Yes	4	99	1.26	.694			
No	11	344					
Diabetes							
Yes	6	129	1.62	.368			
No	9	314					
Hypertension							
Yes	11	317	1.09	.881			
No	4	126					
CAD							
Yes	5	38	5.33	.004	3.63	1.102-11.935	.034
No	10	405					
Transfusion rate							
Yes	3	36	2.83	.120			
No	12	407					

*∗*Preop.: preoperative.

*∗*Cr: creatinine.

*∗*eGFR: estimated glomerular filtration rate.

*∗*Hb: hemoglobin.

*∗*OP time: operative time.

*∗*CKD: chronic kidney disease; *∗*AKD: acute kidney disease.

*∗*CAD: coronary artery disease.

## Data Availability

All the data that support the results can be found in the manuscript.
